# Effects of Hyperbaric Oxygen Therapy on Mitochondrial Respiration and Physical Performance in Middle-Aged Athletes: A Blinded, Randomized Controlled Trial

**DOI:** 10.1186/s40798-021-00403-w

**Published:** 2022-02-08

**Authors:** Amir Hadanny, Yafit Hachmo, Daniella Rozali, Merav Catalogna, Eldad Yaakobi, Marina Sova, Hadar Gattegno, Ramzia Abu Hamed, Erez Lang, Nir Polak, Mony Friedman, Shachar Finci, Yonatan Zemel, Yair Bechor, Noga Gal, Shai Efrati

**Affiliations:** 1 The Sagol Center for Hyperbaric Medicine and Research, Shamir (Assaf-Harofeh) Medical Center, Zerifin, Israel; 2 Sackler School of Medicine, Tel-Aviv University, Tel-Aviv, Israel; 3 Bar Ilan University, Ramat-Gan, Israel; 4 Research and Development Unit, Shamir Medical Center, Zerifin, Israel; 5 Physical Therapy Department, Shamir Medical Center, Zerifin, Israel; 6 Sagol School of Neuroscience, Tel-Aviv University, Tel-Aviv, Israel

**Keywords:** Hyperbaric oxygen therapy, Aging athlete, Athletic training, Oxygen consumption, Mitochondrial function

## Abstract

**Introduction:**

Hyperbaric oxygen therapy (HBOT) has been used to increase endurance performance but has yet to be evaluated in placebo-controlled clinical trials. The current study aimed to evaluate the effect of an intermittent HBOT protocol on maximal physical performance and mitochondrial function in middle-aged master athletes.

**Methods:**

A double-blind, randomized, placebo-controlled study on 37 healthy middle-aged (40–50) master athletes was performed between 2018 and 2020. The subjects were exposed to 40 repeated sessions of either HBOT [two absolute atmospheres (ATA), breathing 100% oxygen for 1 h] or SHAM (1.02ATA, breathing air for 1 h).

**Results:**

Out of 37 athletes, 16 HBOT and 15 SHAM allocated athletes were included in the final analysis. Following HBOT, there was a significant increase in the maximal oxygen consumption (VO2Max) (*p* = 0.010, effect size(es) = 0.989) and in the oxygen consumption measured at the anaerobic threshold (VO2AT)(es = 0.837) compared to the SHAM group. Following HBOT, there were significant increases in both maximal oxygen phosphorylation capacity (es = 1.085, *p* = 0.04), maximal uncoupled capacity (es = 0.956, *p* = 0.02) and mitochondrial mass marker MTG (*p* = 0.0002) compared to the SHAM sessions.

**Conclusion:**

HBOT enhances physical performance in healthy middle-age master athletes, including VO2max, power and VO2AT. The mechanisms may be related to significant improvements in mitochondrial respiration and increased mitochondrial mass.

*Trial Registration* ClinicalTrials.gov Identifier: https://clinicaltrials.gov/ct2/show/NCT03524989 (May 15, 2018).

## Key points

- The current study is the first blinded, randomized controlled trial evaluating the effect of repeated HBOT exposures on physical performance.
- The study indicates that HBOT can enhance physical performance in healthy adults. The main improvements include maximal oxygen consumption, power and the anaerobic threshold.
- By the use of muscle biopsies, it was demonstrated that the mechanisms related to HBOT induce significant increase in mitochondrial respiration and mitochondrial mass.

## Introduction

Maximal aerobic fitness, evaluated by measuring the maximal oxygen capacity (VO2max) and anaerobic threshold (AT), strongly correlates with skeletal muscle mitochondria content (mitochondrial quantity) and skeletal muscle respiratory capacity, i.e., mitochondrial respiration (mitochondrial quality) [1–3]. At the cellular level, athletic training can increase both mitochondrial quality and quantity parallel to the improvements in VO2max and AT, measured during maximal cardiopulmonary exercise tests (CPET) [2, 4]. Moreover, it has been established that mitochondria respiration and density differ between untrained and trained individuals [5, 6].

Several approaches have been suggested to enhance aerobic fitness by inducing mitochondrial adaptions beyond standard physical exercises. One of the commonly used approaches includes training under hypoxic conditions. Due to its systemic and peripheral stimulation, hypoxic training creates metabolic stress at the cellular level, which intensifies mitochondrial adaptations. However, controlled trials demonstrated contradictory results in both mitochondrial respiration and aerobic fitness [7, 8]. The main reason lies in the fact that performance deteriorates during hypoxic exposures because of the low oxygen supply to the muscles. Unlike hypoxia, muscles perform better under hyperoxia and therefore, normobaric hyperoxia may temporarily enhance endurance and sprint interval performances (while breathing oxygen) [9]. However, long-term mitochondrial adaptations and aerobic fitness changes were not observed after standard exposure to normobaric hyperoxia [9].

Hyperbaric oxygen therapy (HBOT) utilizes 100% oxygen in an environmental pressure higher than one absolute atmosphere (ATA). Repeated intermittent hyperoxic exposures have been shown to induce physiological effects which normally occur during hypoxia in a hyperoxic environment [10–13]. Thus, intermittent fluctuations can potentially induce mitochondrial adaptations without the harmful hypoxic environment [14]. Previous evidence shows that hyperbaric oxygen can enhance aerobic performances and increase VO2Max by 10–14% during HBOT exposure [15]. Recently, Burgos et al. demonstrated that 3 weeks of intermittent HBOT sessions increase endurance performances in a pilot study on 12 young soccer players with a moderate effect size on power output and VO2Max [16]. However, the effect of intermittent hyperbaric exposures on performance was never evaluated in a blinded randomized controlled manner.

The aim of the current study was to evaluate the effect of an intermittent HBOT protocol on maximal physical performance and its effect on mitochondrial function in middle-age master athletes.

## Methods

### Study Design

A double-blind, randomized, 1:1 ratio, placebo-controlled study of healthy middle-aged master athletes. The study was done between May 2018 and December 2020 and the protocol was approved by the Shamir Medical Center institutional review board. The study was registered in the National Institutes of Health (NIH) clinical trials registry, number NCT03524989 (30/04/2018). The study was performed in the Shamir Medical Center. All methods were performed in accordance with the relevant guidelines and regulations in accordance with the Declaration of Helsinki.

### Subjects

Thirty-seven healthy master athletes, aged 40–50, who performed aerobic sports at least four times a week at moderate-high performance for their age group, with no significant musculoskeletal injury in the past 3 months, were enrolled. Exclusion criteria included: previous treatment with HBOT for any reason during the last 3 months, debilitating significant musculoskeletal injury, lung pathologies, middle or inner ear pathologies, claustrophobia, chronic illness, chronic medications or active smoking. Athletes were recruited via advertisements and social media. Informed consent was obtained from all subjects.

All the athletes were requested to continue their current training regimen, with no changes in volume or training intensity.

### Randomization and Masking

After signing an informed consent, the athletes were randomly assigned (1:1) to either the HBOT or the SHAM-placebo groups. The randomization code was generated by a nurse coordinator who was masked to the study and was not involved in the execution of the study. Until study closure, the treatment codes were available only to this nurse and the HBOT technicians. Participant enrollment was done by physicians who were masked to the study randomization. Assessors were also blinded to the athletes’ intervention assignment.

To evaluate the blinding, following the first session, the athletes were asked to discreetly answer a two-question questionnaire about their perception of whether they were allocated to the treatment or SHAM group.

All data were stored in a dedicated database and were checked for accuracy and completeness. A masked data review was done before code-breaking and analysis, according to a standard procedure at our unit.

### Interventions

Both the HBOT and SHAM-placebo protocols were conducted in a multiplace Starmed-2700 chamber (HAUX, Germany). Pressure gauges and informative screens within the chamber were disconnected for the athletes’ blinding. The protocol comprised of 40 daily sessions, five sessions per week, within a 2-month period. The athletes were instructed to maintain their usual training program throughout the study.

The HBOT protocol included breathing 100% oxygen by mask at 2ATA for 60 min with no air breaks. Compression/decompression rates were 1 m/min.

The SHAM-placebo protocol included breathing 21% oxygen by mask at 1.02 ATA for 60 min. In order to achieve blinding and have the athletes perform pressure equalization, compression to 1.2 ATA was performed for the first 5 min (0.4 m/min), followed by decompression to 1.02 ATA (0.4 m/min) in the following 5 min. The minimal added 0.02 ATA was mandatory for blinding and for avoiding chamber door opening during the session.

### Outcomes

The athletes were evaluated at baseline, 1–2 weeks prior to their interventional protocol and 1–2 weeks after the last HBOT/SHAM session.

#### Cardiopulmonary Maximal Exercise Test (CPET)

Exercise tests were conducted on an E100 cycle ergometer (COSMED, Rome, Italy). Gas exchange was measured by a Quark CPET system (COSMED), with breath-by-breath sampling technology and integrated heartrate and exercise ECG monitoring and recording with a 12-lead ECG system (COSMED, Rome, Italy). Data were collected on a dedicated computer using the Omnia Metabolic Modules software (COSMED). Before each test, the gas analyzers and flow meter were calibrated. The start of the protocol included a one-minute rest without pedaling, followed by a 2-min warm up. The testing protocol included a ramp power increase of 30 watts every minute starting from 0 W, while pedaling cadence had to be maintained at 70 rpm. Exhaustion was reached when cadence could not be maintained above 70 rpm or when a participant terminated the test. Subsequently, a three-minute recovery with a pedaling workload of 0 W was initiated.

#### Maximal Exercise Tests

A blinded physiologist performed analysis of each CPET test separately, masked from the athletes’ name, group allocation, date of performance and whether the test was a baseline or post-intervention measurement. The breath-by-breath dataset was averaged in epochs of seven breaths and both VO2Max and VO2AT (VO2 at the ventilatory anaerobic threshold) were determined according to classic criteria (based on the plateau in the VO2 plot with increasing workload for VO2Max [17] and minute ventilation (V_E_), respiratory exchange ratio (RER), end-tidal partial pressure of oxygen (PetO2), ratio of minute ventilation to oxygen consumption (V_E_ /VO_2_) and the ratio of minute ventilation to carbon dioxide (V_E_ /VCO_2_) for VO2AT [18]). Compared parameters were maximal power output, maximal oxygen consumption (VO2Max), anaerobic oxygen consumption (VO2AT), breathing reserve (BR), RER, heartrate, V_E_ and volume of CO2 expired (VCO2).

#### Mitochondrial Respiration

Muscle samples were taken from the gluteus maximus by a fine-needle biopsy technique using a TruCut biopsy needle and a 14G puncture cannula after prepping, draping and local anesthesia. The gluteus maximus muscle was chosen to minimize any interference with the athletes’ daily training and increase participation rates. Athletes underwent muscle biopsies at baseline (1–2 weeks prior to intervention) and upon its completion (1–2 weeks post-intervention). Muscle tissue of about 5–10 mg was immediately put in ice-cold biopsy preservation solution (BIOPS). Samples were immediately transferred to saponin solution for membrane permeabilization for 25 min followed by two cycles of 10 min each in respiration medium (MiR05) prior to experimentation. Samples were analyzed by high-resolution respirometry.

Mitochondrial respiration was measured using the Oroboros® Oxygraph-2 K (Oroboros Instruments, Innsbruck, Austria). This device allows to simultaneously record the O_2_ concentration in two parallel chambers, calibrated for 2 ml of respiration medium (MiR05). Mitochondrial respiration was quantified in terms of oxygen flux (*J*O_2_) based on the rate of change of the O_2_ concentration in the chambers normalized for wet tissue volume. Two mg wet weight samples were added to each Oxygraph chamber and normalized to the amount of tissue per chamber.

The titration sequence used for the human muscle samples was as follows: 10 mM pyruvate, 5 mM malate, stepwise titration of 2.5 mM ADP, 10 μM cytochrome c, 10 mM glutamate, 10 mM succinate, stepwise titration of 0.5 μM carbonyl cyanide p-(trifluoromethoxy)-phenylhydrazone (FCCP), 0.5 μM rotenone and 5 μM antimycin A. A measure of the proton leak (*leak*) was obtained following the addition of pyruvate and malate. A stepwise titration of ADP to saturated concentrations allowed us to selectively quantify the activity of complex I (*C*_*I*_) (the oxygen consumption rate through the NADH pathway). Ten μM cytochrome c was added as an internal control to survey the integrity of the outer mitochondrial membrane. Glutamate was added to evaluate its additive effect on CI activity. The maximum oxidative capacity through complex I + II (*maximum OxPhos*) was determined after the addition of the FADH2 pathway substrate succinate. Subsequent injections of the uncoupler FCCP allowed obtaining the maximum respiratory activity in the uncoupled state (*maximum uncoupled*). Finally, the selective complex II activity (*C*_*II*_) was obtained at the end of the titration sequence by adding the complex I inhibitor rotenone in the maximum uncoupled state. In a final step, complex III was inhibited by the administration of antimycin A.

#### Mitochondrial Markers

Muscle samples were taken from the gluteus maximus by a fine-needle biopsy technique (see above) at baseline (1–2 weeks prior to intervention) and upon its completion (1–2 weeks post-intervention). Following membrane permeabilization by saponin, samples were immersed in 4% paraformaldehyde for 6 h and then transferred to 70% ethanol for preservation until paraffin embedding. Due to the small size of the sample following immersion, it was not organized in a specific orientation. Paraffin-embedded sections were de-paraffinized, rehydrated and washed in phosphate-buffered saline (PBS). Antigen retrieval was performed in 1 mM EDTA, pH 8.0 for 40 min. After blocking with 5% normal goat serum (Abcam, ab7481) supplemented by 2% BSA for one hour at RT, sections were incubated with primary antibodies for MNF1/2 (1:100), PGC-1 alpha (Abcam ab54481, 1:200) and OPA1 (Abcam ab157457, 1:300) overnight at 4 °C, followed by a secondary antibody for one hour at 4 °C, and followed by either goat anti-mouse or goat anti-rabbit secondary antibodies (Abcam 1:500) for one hour at RT. The sections were then mounted using Fluoroshield mounting medium with DAPI (ab104139). Control samples were exposed to only the secondary antibody to rule out unspecific staining.

Mitochondrial mass was evaluated with the MitoTracker Green FM dye (Molecular Probes) as previously described [19]. Briefly, following deparaffinization and rehydration, the sections were incubated for 30 min at room temperature with 100 nM MitoTracker Green FM followed by rinses with PBS.

Stained slides were imaged for fluorescence using a Lionheart™ FX Automated Fluorescent Microscope. Total intensity divided by total area in three random fields (for each experimental sample) was measured with Lionheart™ FX Gene 5 image analysis software. Fluorescence intensity values for each experimental group were averaged and presented as mean fluorescent intensity (MFI).

#### Pulmonary Function

Measurements of pulmonary functions were performed using the KoKo Sx1000 spirometer (Nspire Health, USA), 1–2 weeks prior to and after the last HBOT/SHAM session. The equipment was calibrated using a 3–l syringe before performing measurements according to the manufacturer’s instructions. Measurements were performed by a trained technician. The forced expiratory maneuvers were performed as recommended by the guidelines [19].

The forced vital capacity (FVC) forced expiratory volume in 1 s (FEV1), the Tiffeneau–Pinelli index (FEV1/FVC) and peak expiratory flow rate (PEF) were taken as the highest readings obtained from at least three satisfactory forced expiratory maneuvers. Mean forced mid-expiratory flow rate (FEF25–75%) and forced expiratory flow rates at 25, 50 and 75% of FVC expired (FEF25%, FEF50% and FEF75%) were taken as the best values from flow–volume loops not differing by > 5% from the highest FVC.

#### Body Composition

An Inbody720 body composition analyzer (Biospace Co., South Korea) was used to detect the human body composition based on recommendations provided in the user manual. The athletes’ bare feet stood on the pedal plate electrode, hands naturally hanging down and holding the hand electrode gently, and the angle between the trunk and upper limbs was maintained at ~ 15°. Indexes included basal metabolic rate, lean body weight, intracellular fluid, extracellular fluid, body water content, skeletal muscle, body fat, abdominal obesity, etc. Weight was measured in kilograms with the athletes barefoot in minimal clothing by a digital scale (Beurer, Germany), and height was measured in centimeters. BMI was calculated as the weight in kilograms divided by the square of the height in meters (kg/m^2^). Athletes underwent body composition analysis at baseline, 1–2 weeks prior to intervention and upon its completion, and 1–2 weeks post-intervention.

#### Physical Measurements

A trained physical therapist, who was masked from group allocation, performed physical measurements including range of motion, vertical jump, maximal quadriceps power, a step test and an agility test.

#### Safety

Athletes were monitored for adverse events including: barotraumas (either ear or sinuses) and oxygen toxicity (pulmonary and central nervous system).

### Statistical Analysis

Continuous data are expressed as means ± standard-deviation. Normal distributions for all variables were tested using the Kolmogorov–Smirnov test. Unpaired and paired t tests were performed to compare variables between and within the two groups. Net effect sizes were evaluated using Cohen's d method.

Categorical data are expressed in numbers and percentages and were compared by chi-square tests. Univariate analyses were performed using chi-square/Fisher’s exact test to identify significant variables (*P* < 0.05).

To evaluate HBOT’s effects on physical performance, a within-subject repeated measures ANOVA model was used to test the main interaction effect between time and group. To evaluate HBOT’s effects on mitochondrial respiration, a within-subject analysis of covariance (ANCOVA) model was used to test the main interaction effect between time and group. The false discovery rate (FDR) method was used for multiple comparisons correction.

Due to non-normal distribution, mitochondrial mass markers were analyzed using the Mann–Whitney U test. Relative changes (in percentages) were calculated as post-intervention to baseline difference divided by the baseline and multiplied by 100.

#### Sample Size

Sample size was calculated based on Burgos et al. pilot study [16] on 12 young soccer players which showed a moderate effect size on VO2Max. A moderate effect size of 0.3 in VO2Max in a repeated measures ANOVA design, with a power of 85% and an alpha of 5%, requires 14 athletes in each arm. Adding a 15% dropout rate would require 32 subjects in total.

## Results

**Fig. 1:**
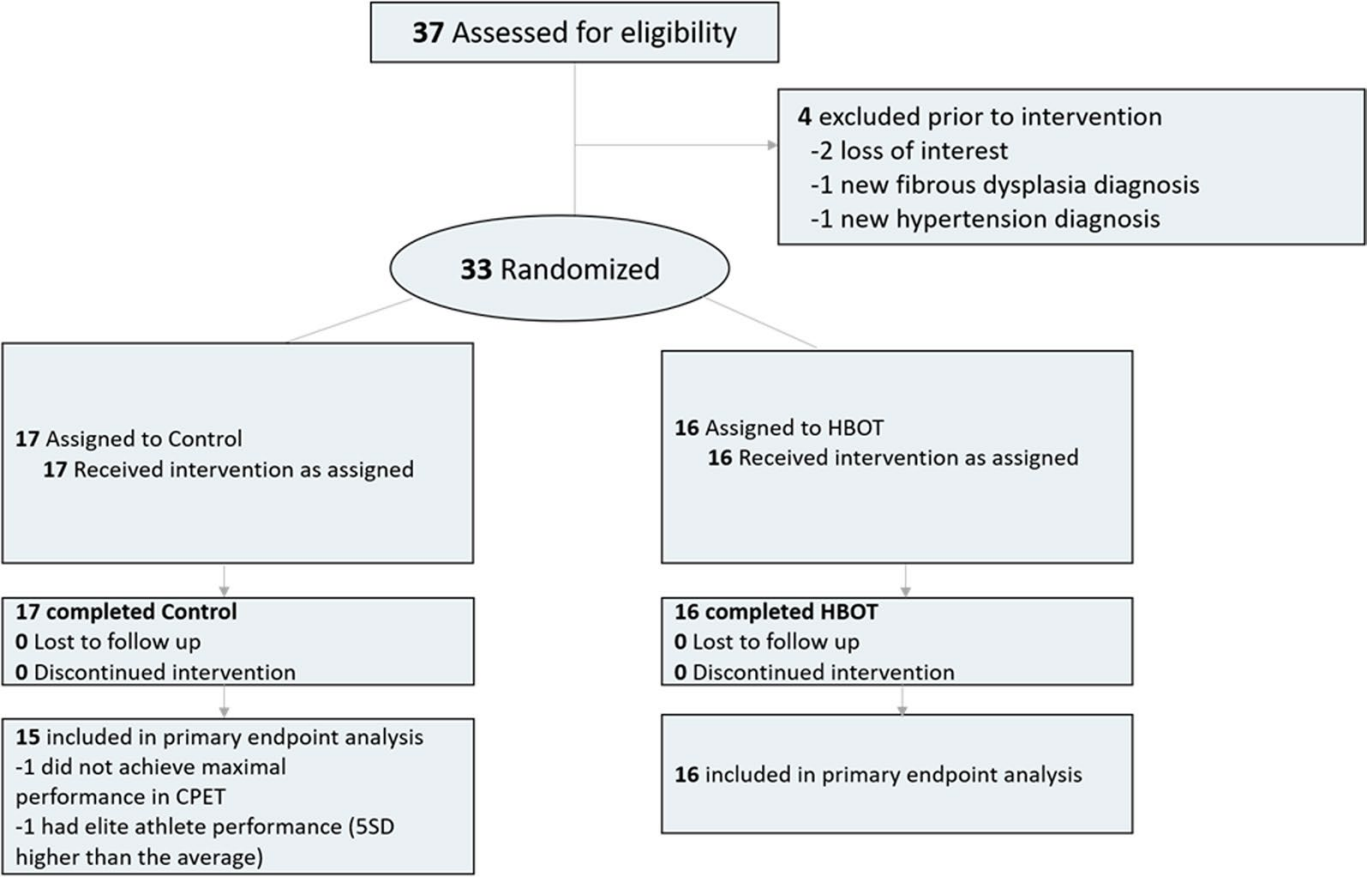
Athletes’ flowchart

Thirty-seven athletes signed the informed consent form. Two athletes quit the study during baseline evaluations. Two athletes were excluded following baseline evaluations due to newly diagnosed hypertension and skull fibrous dysplasia. Two additional athletes were excluded from the final analysis; one participant did not achieve maximal exercise in his baseline CPET, and the other had a VO2Max which was five standard deviations higher than the rest of the athletes (Fig. 1). The baseline characteristics and comparability of the cohort are provided in Table 1.

**Table 1:** Athletes' characteristics

	All (*N* = 31)	SHAM (*N* = 15)	HBOT (*N* = 16)	Significance
Age (years)	44.1 ± 2.96	44.60 ± 2.87	43.63 ± 3.05	0.368
Males	26 (83.9%)	12 (80.0%)	14 (87.5%)	0.65F
Experience	10.97 ± 6.30	11.33 ± 6.88	10.63 ± 5.90	0.76
Sport type				0.02
Triathlon	10 (32.3%)	3 (30%)	7 (43.8%)	
Running	13 (41.9%)	4 (26.7%)	9 (56.3%)	
Swimming	2 (6.5%)	2 (13.3%)	0	
Cycling	3 (9.7%)	3 (20%)	0	
Other	3 (9.7%)	3 (20%)	0	
Previous injuries	6 (19.4%)	2 (33.3%)	4 (25%)	0.65F

Baseline athletes’ parameters are summarized in Table 1. There was no difference between the groups as to any of the baseline characteristics except for training type. In the HBOT arm, all athletes were runners or triathletes, whereas in the SHAM-control group there were also swimmers, cyclists and others (*p* = 0.02).

### Blinding Evaluation

**Fig. 2:**
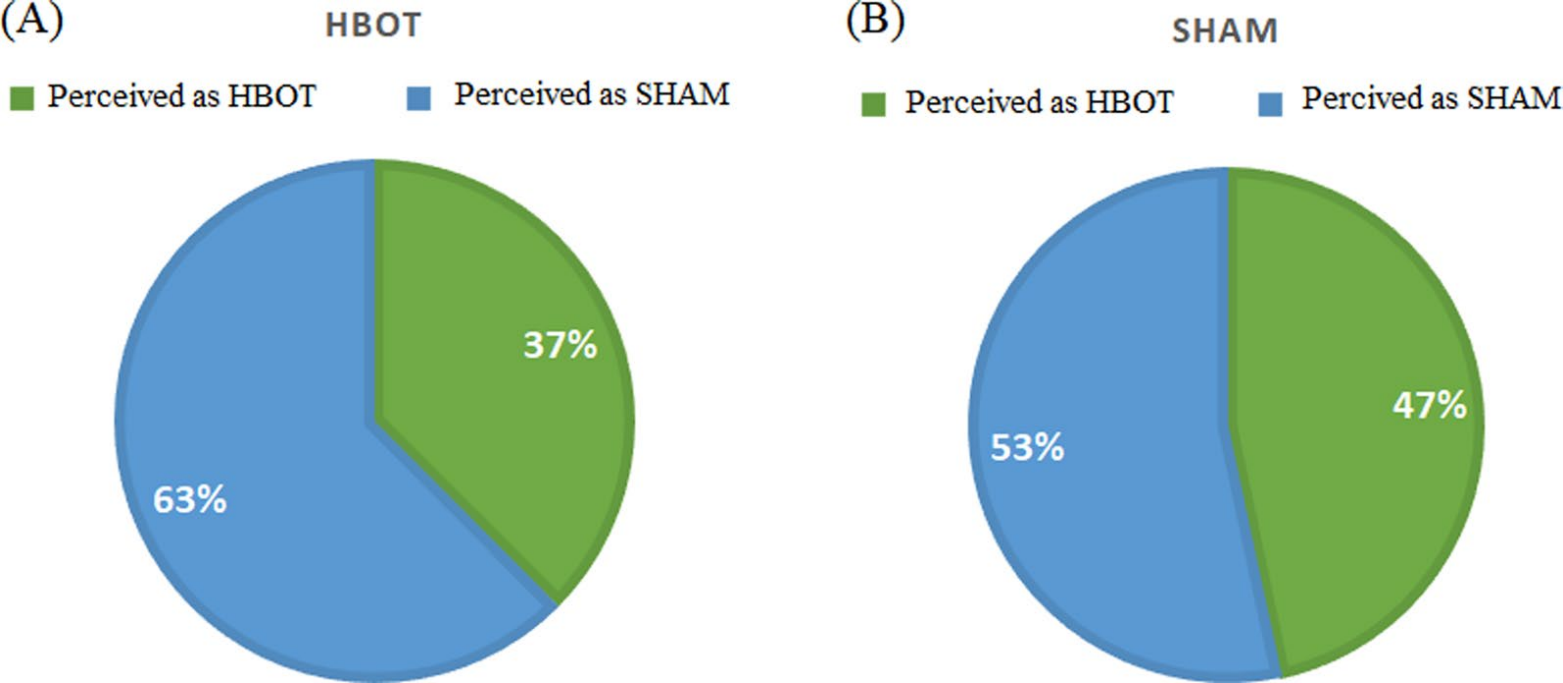
SHAM protocol quality control. Rates of group allocation perception by the HBOT group subjects (**A**) and SHAM group subjects (**B**)

Athlete blinding was achieved, where 63% of HBOT group perceived their sessions as SHAM and 53% of the SHAM group perceived their sessions as SHAM (*p* = 0.60) (Fig. 2).

### Physical Performance Evaluation

There were no significant differences between the groups in CPET parameters at baseline. Following HBOT, there was a significant increase in the maximal oxygen consumption (VO2Max) (2834.50 ± 556.65 ml/min to 2956.19 ± 540.85 ml/min, group X time interaction *F* = 7.57, *p* = 0.010) with a large net effect size of 0.989 [0.210–1.76] compared to the SHAM group (Table 2). Similar results were noted in VO2/kg (Table 2). Oxygen consumption measured at the anaerobic threshold (VO2AT) was significantly increased in the HBOT group (1196.56 ± 218.29 ml/min to 1326.56 ± 249.11 ml/min, group X time *F* = 5.43, *p* = 0.026) with a large net effect size of 0.837 [0.071–1.604], compared to the SHAM group (Table 2).

**Table 2:** CPET changes

	SHAM-Placebo (*N* = 15)			HBOT (*N* = 16)					
	Baseline	SHAM	*P* value	Baseline	Post-HBOT	*P* value	Baseline comparison *P* value	Net effect size	Time X group interaction F(P)
*Primary endpoint*									
VO2Max (mL/min)	2673.33 ± 748.03	2631.07 ± 751.31	0.14	2834.50 ± 556.65	2956.19 ± 540.85	**0.03**	0.49	0.989 [0.210–1.76]	7.57(0.010)*
VO2AT (mL/min)	1210.87 ± 239.80	1208.53 ± 266.18	0.93	1196.56 ± 218.29	1326.56 ± 249.11	**0.01**	0.86	0.837 [0.071–1.604]	5.43(0.026)*
*Secondary endpoints*									
Power (watts)	256.27 ± 66.43	254.73 ± 68.25	0.78	277.37 ± 46.14	290.93 ± 51.87	0.004*	0.31	0.808 [0.04–1.57]	5.05(0.03)*
VO2/Kg (mL/kg/min)	36.08 ± 9.08	35.51 ± 8.85	0.15	42.00 ± 9.80	43.86 ± 9.04	**0.02**	0.09	1.019[0.238–1.800]	8.04(0.008)*
BR (%)	32.64 ± 12.42	36.77 ± 12.05	0.14	35.71 ± 13.07	30.67 ± 15.05	**0.05**	0.508	− 0.91[− 0.168–(− 0.140)]	6.44(0.016)*
VO2AT/VO2Max (%)	46.95 ± 8.98	47.25 ± 7.32	0.77	42.48 ± 4.33	45.46 ± 8.29	0.15	0.08	0.424 [− 0.138–1.167]	1.39 (0.247)

VO2MAX = maximal oxygen consumption, VO2AT = oxygen consumption at the anaerobic threshold, Kg = Kilograms, BR = breathing reserve

*P* < 0.05 in bold, *Significant following false discovery rate corrections

The HBOT group demonstrated a significant increase in maximal power compared to the SHAM group, with a large net effect size of 0.808 [0.04–1.57] (group X time interaction *F* = 5.05, *p* = 0.03) (Table 2). In addition, compared to the SHAM group, there was a significant decrease in breathing reserve in the HBOT group with a large net effect size − 0.91[− 0.168–(− 0.140)], (group X time interaction *F* = 6.44, *p* = 0.016) (Table 2).

No significant differences were noted in body composition and pulmonary function measurements between the groups at baseline and following HBOT/SHAM (Table 3). In addition, there were no significant differences in range of motion, upper or lower limbs power, vertical jump or agility (Table 3).

**Table 3:** Pulmonary function and body composition

	SHAM-Placebo (*N* = 16)			HBOT (*N* = 16)					
	Baseline	SHAM	3 months	Baseline	Post-HBOT	3 months	Baseline comparison *P* value	Net effect size	Time X group interaction F(P)
			*P* value			*P* value			
*Pulmonary function*									
FEV1 (liters)	3.79 ± 0.48	3.89 ± 0.57	0.29	3.46 ± 0.37	3.42 ± 0.40	0.64	0.08	0.469 [− 0.435–1.375]	1.16 (0.29)
FVC1 (liters)	4.71 ± 0.79	4.91 ± 0.71	0.09	4.21 ± 4.27	4.27 ± 0.49	0.61	0.11	0.404 [− 0.462–1.271]	0.77(0.38)
FEV1/FVC	0.80 ± 0.04	0.79 ± 0.05	0.45	0.82 ± 0.08	0.81 ± 0.05	0.31	0.30	0.437 [− 0.431–1.305]	0.42(0.52)
FEF25-75 (liters)	3.59 ± 0.85	3.64 ± 0.83	0.77	3.77 ± 1.05	3.29 ± 0.62	0.12	0.66	0.751 [− 0.136–1.638]	3.29(0.08)
*Body composition*									
Weight (kilograms)	73.58 ± 11.34	73.09 ± 12.02	0.21	68.46 ± 6.70	67.91 ± 6.60	0.16	0.13	− 0.092 [− 0.828–0.642]	0.01(0.91)
BMI	24.16 ± 2.42	23.94 ± 2.62	0.10	23.21 ± 2.53	23.01 ± 2.47	0.16	0.29	0.120 [− 0.614–0.856]	0.03(0.86)
BFM (kilograms)	14.2 ± 4.67	13.71 ± 5.19	0.13	11.33 ± 4.66	10.43 ± 4.39	**0.04**	0.09	0.030 [− 0.704–0.765]	0.60(0.44)
SMM (kilograms)	33.51 ± 5.63	33.55 ± 5.63	0.85	32.16 ± 3.73	32.4 ± 3.75	0.11	0.43	− 0.214 [− 0.951–0.522]	0.58(0.44)
TBW (liters)	43.48 ± 6.64	43.42 ± 6.69	0.84	41.78 ± 4.60	42.03 ± 4.65	0.21	0.41	− 0.133 [− 0.869–0.602]	0.75(0.39)
ICW (liters)	27.23 ± 4.32	27.27 ± 4.31	0.82	26.19 ± 2.87	26.38 ± 2.86	0.08	0.43	− 0.226 [− 0.926–0.510]	0.55(0.46)
*Physical measurements*									
Knee ROM (degrees)	61.45 ± 12.99	62.75 ± 15.85	0.62	61.77 ± 11.77	59.69 ± 13.55	0.48	0.94	0.327 [− 0.456–1.111]	0.74 (0.39)
Quads max power (kilograms)	20.9 ± 7.32	18.7 ± 7.59	0.07	22.34 ± 5.40	21.66 ± 5.36	0.66	0.55	− 0.296 [− 1.079–0.486]	0.61 (0.44)
Vertical jump (centimeters)	39.27 ± 8.42	38.38 ± 5.43	0.53	40.1 ± 7.85	40.49 ± 8.89	0.68	0.79	− 0.297[− 1.081–0.485]	0.62 (0.44)
Step test (beats/minute)	135.69 ± 15.61	127.07 ± 17.59	**0.012**	136.13 ± 20.62	133.42 ± 16.57	0.54	0.95	− 0.626[− 1.438–0.186]	2.64 (0.11)
Agility test (seconds)	15.99 ± 1.69	16.25 ± 2.07	0.85	15.86 ± 1.16	15.40 ± 1.62	0.36	0.81	0.251 [− 0.549–1.052]	0.42 (0.52)

FEV1 = forced expiratory volume in one second, FVC = forced vital capacity, FEF25-75 = percentage of the predicted value for forced expiratory flow at 25–75% of forced vital capacity, BMI = body mass index, BFM = body fat mass, SMM = skeletal muscle mass, TBW = total body water, ICW = intracellular water

*P* < 0.05 in bold, *Significant following false discovery rate corrections

### Mitochondrial Respiration Evaluation

Thirty athletes from those included in the final analysis consented to muscle biopsies. Three athletes refused to undergo their second biopsy and five athletes’ biopsies did not pass quality control for respiration analysis. Thus, mitochondrial respiration was assessed in 10 HBOT athletes and 12 SHAM athletes. Since there were significant differences between the groups in the baseline mitochondrial respiration parameters, an ANCOVA was performed.

Following HBOT, there were significant increases in both maximal oxygen phosphorylation capacity (effect size 1.085 [0.129–2.041], group X time ANCOVA = 4.89, *p* = 0.04 uncorrected) and maximal uncoupled capacity (0.956 [0.013–1.898], group X time ANCOVA *F* = 6.04, *p* = 0.02, uncorrected) compared to the SHAM sessions. In addition, mitochondrial complex I function increased significantly (effect size = 1.120 [0.160–2.080], group X time ANCOVA *F* = 7.31, *p* = 0.01 uncorrected for multiple comparisons) in the HBOT group compared to the SHAM group. No changes were noted in the mitochondrial complex II uncoupled activity and proton leak rate (Table 4).

**Table 4:** Mitochondrial respiration changes

	SHAM-Placebo (*N* = 12)			HBOT (*N* = 10)					
	Baseline	SHAM	3 months	Baseline	Post-HBOT	3 months	Baseline Comparison *P* value	Net effect size	ANCOVA (P)
			*P-*value			*P* value			
*Primary endpoint*									
Leak [pmol /(s*mg)]	8.51 ± 3.26	7.34 ± 3.92	0.15	9.72 ± 1.90	11.98 ± 5.60	0.17	0.32	0.429 [− 0.47–1.33]	3.80 (0.06)
Complex I (Pyruvate–Malate–Glutamate) [pmol /(s*mg)]	43.14 ± 16.03	35.21 ± 16.37	**0.03**	58.05 ± 16.05	63.75 ± 19.99	0.21	**0.04**	1.120 [0.160–2.080]	**7.31 (0.01)**
Complex II (uncoupled) [pmol /(s*mg)]	44.03 ± 13.13	46.32 ± 10.10	0.49	61.32 ± 14.39	63.19 ± 14.40	0.65	**0.008**	1.223 [0.250–2.195]	2.19 (0.15)
Maximal coupled capacity (Succinate)	74.78 ± 20.40	72.22 ± 21.23	0.66	97.85 ± 22.21	105.56 ± 21.61	0.26	**0.019**	1.085 [0.129–2.041]	**4.89 (0.04)**
[pmol /(s*mg)] Maximal uncoupled capacity [pmol /(s*mg)]	87.94 ± 22.99	80.36 ± 23.81	0.24	117.30 ± 37.96	121.49 ± 29.51	0.56	**0.036**	0.956 [0.013–1.898]	**6.04 (0.02)**

*P* < 0.05 in bold, *Significant following false discovery rate corrections

### Mitochondrial Mass Evaluation

Twelve (6 HBOT, 6 SHAM) out of the 22 athletes with available muscle biopsies at baseline and post-intervention (see above) were evaluated for mitochondrial mass. In all other athletes, the muscle biopsy sample size was limited and used entirely for mitochondrial respiration evaluation, see above).

**Fig.3:**
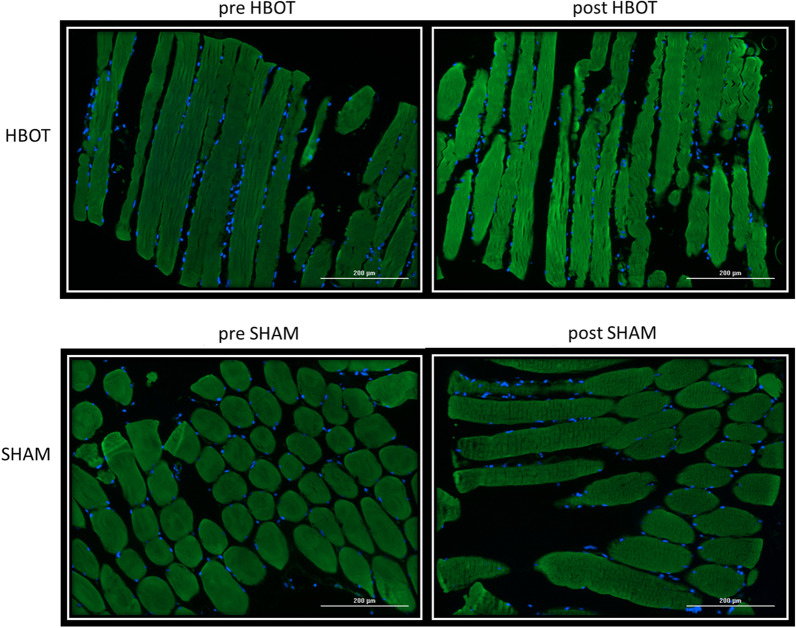
Mitochondrial mass changes. High-resolution micrograph of muscle staining with MTG demonstrates a significant increase in the mitochondrial mass marker MTG compared to the SHAM group (17.12% ± 20.2 vs. − 8.54 ± 8.41, *p* = 0.0002). Positive counts are represented by green dots. Greener/brighter images reflect higher counts of MTG staining

Following HBOT, there was a significant increase in the mitochondrial mass marker MTG compared to the SHAM group (17.12% ± 20.2 vs. − 8.54 ± 8.41, *p* = 0.0002) (Table 5, Fig. 3). There were no significant changes in the biogenesis marker PGC1alpha (*p* = 0.699) and fusion markers OPA1 (*p* = 0.12) and MNF1 + 2 (*p* = 0.09) (Table 5).

**Table 5:** Mitochondrial markers changes

	SHAM-Placebo (*N* = 5)			HBOT (*N* = 6)			Sig
	Baseline	SHAM	Relative change	Baseline	Post-HBOT	Relative change	
*Mitochondrial marker*							
PGC1alpha (counts)	16,090.8 ± 4651.7	14,243.8 ± 2935.3	(− 9.49) ± 13.25	13,380.0 ± 3500.4	14,315.5 ± 5606.3	10.21 ± 48.66	0.699
MTG (counts)	44,117.0 ± 106,170	40,350.2 ± 10,484.4	(− 8.54) ± 8.41	43,268.0 ± 10,650.8	49,472.5 ± 7773.2	17.12 ± 20.2	0.002*
MNF1 + 2 (counts)	38,931.3 ± 16,182.7	35,007.5 ± 10,945.1	(− 5.43) ± 18.17	38,685.5 ± 11,056.6	44,766.0 ± 14,288.5	16.17 ± 22.59	0.09
OPA1 (counts)	44,246.6 ± 20,029.1	44,697.7 ± 22,486.5	(− 0.93) ± 6.60	31,903.4 ± 10,447.99	38,031.2 ± 13,043.2	20.04 ± 27.54	0.12

*P* < 0.05 in bold, *Significant following false discovery rate corrections

### Safety and Side Effects

During the study, two subjects in the HBOT group developed an upper airway viral infection. In the SHAM group, two subjects developed pneumonia. One participant developed a gluteal subcutaneous hematoma following the second biopsy and was treated conservatively with full recovery. No other adverse events were reported.

## Discussion

This study shows that HBOT may significantly enhance physical performance beyond training in healthy master athletes. Both VO2Max, VO2AT and power significantly increased following intermittent HBOT exposures compared to the SHAM exposures. Moreover, for the first time in humans using muscle biopsies, we demonstrated that HBOT may directly improve mitochondrial respiration and increase mitochondrial mass.

Endurance training-mediated changes in cardiorespiratory fitness level are most frequently quantified via VO2max and AT. Previous studies have shown different exercise programs (such as intervals, high intensity and others) can increase VO2max with significantly large effect sizes (0.5–2.0) [20]. Notably, the control groups in those studies were other exercise training regimens and did not include SHAM exposures or training. In the current study, the control group underwent a SHAM procedure, blinded to allocation as confirmed by questioner. Since all participants were blinded to intervention allocation and were not instructed to modify their training, any changes in their daily/exercise routine, if were, and any changes in the study end points could be attributed only to the investigated intervention. For the first time in humans, it is demonstrated that VO2max increased significantly by HBOT as compared to SHAM treatment. One can anticipate that combining high-intensity interval training and HBOT will have synergistic effects but that remains to be investigated.

AT is considered a better predictor of performance than VO2max, with higher AT indicating higher sustained intensity without producing acidosis and the associated lactate accumulation [21]. Additionally, during the CPET test, AT can be measured objectively regardless of the participant's motivation which may affect VO2peak [22]. We used the ventilatory AT, as it is a good estimation of blood lactate concentrations, avoiding the need of multiple needle pricks and longer testing protocols during CPET [18]. It was found that HBOT induces a prominent improvement effect on oxygen consumption measured at the anaerobic threshold.

The results of the current study correlate with the physiological understandings gathered from well-designed pre-clinical studies. In a well-designed mice model study, it was demonstrated that adding intermittent hyperbaric exposure to exercise training further improves endurance performance by facilitating oxidative and glycolytic capacities and by increasing the expression of proteins involved in mitochondrial biogenesis in striated muscles [23, 24]. In humans, Decato et al. showed that combining six intermittent HBOT sessions to an exercise training regimen induces better cardiorespiratory fitness compared to exercise training alone [25]. In this study, for the first time in humans, we demonstrated that HBOT enhances aerobic fitness in a randomized sham-controlled manner.

An athlete's endurance performance is determined by the VO2max, AT and the exercise efficiency [26, 27]. Mitochondrial oxidative phosphorylation capacity of the skeletal muscle appears central to all three physiological components. An individual's VO2Max is limited by cardiac output, muscle blood flow and the blood’s oxygen carrying capacity [26]. There is also a strong linear correlation between VO2Max and mitochondrial volume density and capillary density [28]. The anaerobic threshold is parallel to the mitochondrial capacity of the skeletal muscle [29]. Additionally, skeletal muscle oxidative capacity also correlates strongly with exercise efficiency [30]. Thus, it has been shown that the increase in skeletal muscle respiration capacity that parallels aerobic fitness can be explained by both mitochondria number and mitochondrial respiration [3]. In our study, both mitochondrial respiration and mitochondrial mass increased significantly in parallel to aerobic fitness, as observed by VO2Max, following HBOT compared to the SHAM group.

Previous studies have shown that humans exploit 90% of their mitochondrial capacity while performing maximal exercise at VO2Max [31]. Thus, acute exposure to normobaric or hyperbaric hyperoxia, can increase VO2Max temporarily, if the subject is breathing increased oxygen levels which is usually impractical during real-world exercises/sports. However, this temporary effect would not induce long-term mitochondrial adaptations neither in number nor in respiration. In our study, athletes underwent multiple intermittent hyperbaric exposures, which enabled the mentioned cellular changes. The fact that both CPET and mitochondrial biopsies were done more than 1 week after the last HBOT session further supports the conclusion that repeated HBOT induces significant biological changes, and the beneficial effect is not related to the transient increase in oxygen delivery.

Hypoxic training has been suggested to enhance aerobic fitness with long-term effects. Hypoxia, indeed, induces the hypoxic-induced factor-1α (HIF1α) transcription factor, which initiates a cascade of reactions to cope with the new energetic crisis of low oxygen [14]. However, due to the continuous insufficient level of oxygen, the number of mitochondria is reduced (less generation of new mitochondria) and mitochondrial respiration is inhibited to adapt and preserve life [32]. In comparison, during repeated intermittent hyperbaric exposures, the relative changes in oxygen availability rather than the constant hypoxia/hyperoxia have a more dominant effect on HIF1α induction [10, 33–36]. HIF1α is increased and promotes mitochondrial biogenesis and mitochondrial respiration changes, which are possible as oxygen supplies remain at normal or super-normal states [14]. This phenomenon has been referred as “the hyperoxic–hypoxic paradox,” i.e., increased HIF and its associated cascade at hyperoxic states [14]. Thus, intermittent hyperbaric exposures can be compared to intense interval training which induces mitochondrial adaptations in both number and respiration. Our study shows for the first time in humans, that both mitochondrial respiration (both coupled and uncoupled) and mass are enhanced following HBOT, compared to no changes in the SHAM group.

PGC-1α is a critical regulator of mitochondrial biogenesis in skeletal muscle and promotes this process in response to exercise to maintain a balance between energy need and energy supply. Previous studies have suggested that HBOT increases PGC-1 following repeated exposures [23]. MNF1/2 are involved in the outer mitochondrial membrane fusion while OPA1 mediates fusion of the inner mitochondrial membrane [37]. MNF2 is associated with reduced mitophagy, leading to the accumulation of damaged mitochondria. OPA1 is a sensor of physical activity and decreases with aging-related changes [38]. Although all three increased in the HBOT group, they were not statistically significant. These findings may be explained by the small sample size (*n* = 12) of available muscle biopsies. These results may also be related to the timing of the muscle biopsies. Taken 2 weeks following the last session, active biogenesis may have ended, and mitochondrial mass has increased. MTG enables accurate measurements of mitochondrial mass independent of the mitochondrial function and respiration [39]. In the current study, the HBOT group subsample had increased mitochondrial mass compared to the SHAM group subsample.

The current study has several limitations. First, the relatively small sample size has to be considered, possibly causing decreased sensitivity. However, the presence of significant changes following strict statistical analyses in a small group is indicative of the relatively high potency of the intervention. Second, the duration of the effect is yet to be determined in long-term follow-up studies. Third, the protocol was 40 sessions of 100% oxygen at two ATA HBOT exposure. However, the optimal number of sessions and protocol of each session remains to be determined. Lastly, there were differences in baseline mitochondrial respiration between the two groups which may be related to the heterogenicity of the participants athletes. Mitochondrial respiration variability is expected and has been reported within healthy adults [31, 40]. In the current study, we mitigated these differences using ANCOVA.

## Conclusions

The study indicates that HBOT can enhance physical performance in healthy master athletes. The main improvements include maximal oxygen consumption, power and the anaerobic threshold. By the use of muscle biopsies, it was demonstrated that the mechanisms related to HBOT induce significant improvement in mitochondrial respiration and increase mitochondrial mass.

## Data Availability

The datasets used and/or analyzed during the current study are available from the corresponding author on reasonable request.

## Abbreviations

VO2max: Maximal oxygen capacity
AT: Anaerobic threshold
CPET: Maximal cardiopulmonary exercise tests
HBOT: Hyperbaric oxygen therapy
ATA: Absolute atmosphere
NIH: National Institutes of Health
V_E_: Minute ventilation
RER, PetO2: Ratio of minute ventilation to oxygen consumption (V_E_ / VO_2_)
BR: Breathing reserve
RER: Respiratory exchange ratio
VE: Minute ventilation
VCO2: Volume of CO2 expired
PetO2: End-tidal partial pressure of oxygen
*J*O_2_: Oxygen flux
*C*_*I*_: Oxidative capacity through complex I
maximum OxPhos: Oxidative capacity through complex I + II
*C*_*II*_: Oxidative capacity through complex II
PBS: Phosphate-buffered saline
MFI: Mean fluorescent intensity
FVC: Forced vital capacity
FEV1: Forced expiratory volume in 1 s
PEF: Peak expiratory flow rate
FEF25–75%: Forced expiratory flow at 25–75% of forced vital capacity
FEF25%: Forced expiratory flow rates at 25 of FVC expired
FEF50%: Forced expiratory flow rates at 50 of FVC expired
FEF75%: Forced expiratory flow rates at 75 of FVC expired
ANOVA: Analysis of variance
ANCOVA: Analysis of covariance
FDR: False discovery rate
HIF1α: Hypoxic-induced factor-1α
BMI: Body mass index
BFM: Body fat mass
SMM: Skeletal muscle mass
TBW: Total body water
ICW: Intracellular water
